# Patient Satisfaction in Neurosurgery Clinic

**DOI:** 10.7759/cureus.53176

**Published:** 2024-01-29

**Authors:** Alice S Wang, James G Wiginton, Theo Tran, Paulino Yanez, Christopher King, Dan E Miulli

**Affiliations:** 1 Neurosurgery, Riverside University Health System Medical Center, Moreno Valley, USA; 2 Neurosurgery, Arrowhead Regional Medical Center, Colton, USA; 3 Neurosurgery, California University of Science and Medicine, Colton, USA; 4 Neurosurgery, Kaiser Fontana Medical Center, Fontana, USA; 5 Neurosurgery, Riverside University Health System Medical Center, Colton, USA

**Keywords:** quality improvement research, survey, outpatient clinic, patient satisfaction score, neuro-surgery

## Abstract

Background: National commercial surveys are used to assess patient satisfaction. However, the information obtained does not always correspond to the clinical situation and therefore may be inadequate to help improve a specific patient experience when through no fault of its design, results in low response rates and inadequate specifics.

Objective: The objective is to investigate patient satisfaction using real-time in-person patient experience survey responses at the end of a neurosurgical clinic visit and review the results from these survey responses and those from national commercial survey responses provided by the hospital for the ability to affect change.

Methods: This is a prospective study from October 2023 to December 2023 during which a paper copy of 10 questionnaires derived from a national commercial outpatient clinical survey was given to every unique patient who was neurologically capable of filling it out at the end of his or her neurosurgery clinic visit. The electronic medical record was used to collect patient demographics and details of the clinic visit. National commercial survey responses from July 2022 to November 2023 provided by the hospital were reviewed.

Results: A total of 149 patients were seen in the neurosurgery clinic from October 2023 to December 2023, 121 patients were given the in-person patient satisfaction survey, and the response rate was 100%. The mean age was 46.5 years with females constituted 45.5% of the patient sample. The visit type included 46 (38.0%) new patients, 53 (43.8%) returning patients, and 22 (18.2%) post-op patients, of which 45.5% presented with cranial pathologies. Comparing the patient satisfaction level between those seen by one provider and those seen by two providers, such as resident, or mid-level with attending, patients seen by two providers were less satisfied with “feeling respected by the providers” (4.92 vs. 4.64, p=0.0088), “feeling listened to by the providers” (4.84 vs. 4.50, p=0.0180), and “feeling appreciated that the providers discussed illness prevention” (4.72 vs. 4.29, p=0.0232). Due to a lack of necessary information from our national commercial outpatient clinic survey responses provided by the hospital, a direct comparison between the in-person survey and our national commercial outpatient clinic survey was not made.

Conclusions: Patient satisfaction surveys when not given in real-time in-person run the risk of low response rate and lack of specifics to help guide providers in quality improvement. Our data supports the use of real-time in-person patient satisfaction surveys that not only increase response rate but also provide useful information to help improve patient experience.

## Introduction

Patient satisfaction surveys help communicate patient experience and provide insight for quality improvement. The commonly used surveys measure frequency meaning how often a service was performed and how well a service was performed [1]. The results from these surveys have been used by providers, policymakers, and healthcare administrators to not only evaluate overall hospital and clinic performance but also implement interventions.

However, national commercial outpatient clinic surveys are not without flaws and the validity of these surveys has been studied. Tyser et al. found that only 16.5% of the patients seen in outpatient orthopedic clinics responded to the survey, demonstrating a low response rate, and evidence of non-response bias. The low response rate may be due to patients not checking or responding to their emails which was the avenue this study used to send out surveys [2]. Orthopedic clinic is not the only specialty that suffers from a low response rate. In an ambulatory surgical center, where patients underwent upper extremity surgical procedures, 13.5% of the patients responded to the national commercial survey and 86.5% of the patients were non-responders. The authors also found the responders were older, college-educated, employed, married, and associated with higher incomes [3]. Given the low response rate, the responses may not be representative of the whole patient population.

Moreover, the national commercial outpatient clinic patient satisfaction survey questions are designed to evaluate the patient experience with their healthcare providers, nursing staff, access, and moving through the patient visit [4]. North et al. investigated whether providers can use the responses from national commercial patient surveys to help improve their interactions with the patients and found that the responses failed to provide adequate information for each provider with patient experience improvement [5].

On the other hand, patients may use the internet to gauge which hospitals to go to. The U.S. News and World Report published the scorecard for neurology and neurosurgery at Arrowhead Regional Medical Center and showed that patient experience was below average [6]. This was surprising given most of the patients reported to us that they were very satisfied with the care received. Nonetheless, publicly available information such as the U.S. News and World Report or Healthgrades may attract patients to seek medical services or deflect them away [6,7].

The purpose of this study was to investigate patient satisfaction using real-time, in-person surveys in the neurosurgery clinic and review the results from these survey responses and those from our national commercial outpatient clinic survey responses provided by the hospital.

## Materials and methods

This prospective study received institutional review board approval (Arrowhead Regional Medical Center Protocol 23-50: Patient Satisfaction in Neurosurgery Clinic) and was conducted in the neurosurgery clinic setting. From October 2023 to December 2023, all patients seen in the neurosurgery clinic were eligible to participate in the real-time, in-person patient satisfaction survey. Only patients neurologically capable of filling out the survey were given the survey. Patients unaware of their surroundings or in a coma were excluded from the study. At the end of the clinic visit, a provider walked into the room with a paper copy of the survey in English or Spanish, discussed the purpose of the survey, and obtained written consent from the patient. The provider then left the room to allow the patient to fill out the survey privately. The patient was informed to leave the survey inside the room, and the survey was collected by the front desk/rooming staff and returned to the provider. The questionnaire consisted of ten questions similar to the national outpatient clinical survey. Each question was rated on a Likert-type scale of 5 responses ranging from strongly agree, agree, neutral, disagree, or strongly disagree. Strongly agree was assigned a score of 5, agree was a score of 4, neutral was a score of 3, disagree was a score of 2, and strongly disagree was a score of 1. Patient demographics including age, sex, race, visit type, and pathology were collected from electronic medical records. GraphPad Software was used to analyze the data (GraphPad Software, Boston, MA) [8]. A P-value of < 0.05 will be considered significant.

Responses from our national commercial outpatient clinic patient experience survey from July 2022 to November 2023 were obtained from our hospital’s patient experience department. The staff was queried for knowledge about the national commercial outpatient survey results and whether a change was made. No staff member identified any change that was needed specific to them and therefore, no staff made any changes. An attempt was then made to determine whether a needed change in behavior could be identified by staff from our real-time, in-person survey. Patient demographics, specific types of visits, type of providers, specific providers, and individual responses were not available; therefore, a direct comparison of our national commercial outpatient clinic survey and real-time in-person survey using statistics was not made.

## Results

From October 2023 to December 2023 (seven weeks total), a total of 149 unique patients were seen in the neurosurgery clinic, 121 patients were neurologically capable of filling out the surveys, and all 121 patients completed the surveys (100% response rate). The mean age was 46.5 years and 45.5% of the patients were female. The visit type included 46 (38.0%) new patients, 53 (43.8%) returning patients, and 22 (18.2%) postoperative patients with 45.5% presenting with cranial pathologies (Table 1).

**Table 1:** Patient characteristics (N = 121) Data are presented in N, mean (SD) and %. Abbreviations: N, number of patients; SD, standard deviation

Age, mean years (SD)		46.5 (15.4)
Sex, N (%)	Female	55 (45.5%)
	Male	66 (54.5%)
Race, N (%)	Caucasian	28 (23.1%)
	Black	16 (13.2%)
	Asian	7 (5.8%)
	Other	70 (57.9%)
Visit type, N (%)	New	46 (38.0%)
	Returning	53 (43.8%)
	Post op	22 (18.2%)
Pathology, N (%)	Craniotomy	55 (45.5%)
	Spine	66 (54.5%)

The mean score for each question on the survey is shown in Table 2. On average patients were most satisfied with feeling respected by the provider (mean score of 4.88) and least satisfied with feeling the provider’s knowledge of his or her medical history (mean score of 4.64). There was no difference in patient satisfaction with respect to patient status (new vs. returning vs. post-op) (Table 3). Patients who presented with spine complaints were less satisfied with the front office staff (4.87 vs. 4.68, p=0.0284) (Table 4). Comparing the patient satisfaction level between those seen by one provider and those seen by two providers such as attending resident, or midlevel, patients seen by two providers were less satisfied with feeling respected by the providers (4.92 vs. 4.64, p=0.0088), feeling listened to by the providers (4.84 vs. 4.50, p=0.0180), and feeling appreciated that the providers discussed illness prevention (4.72 vs. 4.29, p=0.0232) (Table 5). There was no difference in patient satisfaction with respect to disposition (follow-up vs. returned as needed vs. surgery scheduled) (Table 6). The majority of the patients were scheduled for follow-up with additional images before follow-up visits (Table 7).

**Table 2:** Patient satisfaction survey outcomes Data are presented in mean (SD). Abbreviations: N, number of patients; SD, standard deviation.

Score per question, lowest 1 to highest 5 (N = 121)	Mean (SD)	Top answer score
I feel the provider shows respect to me.	4.88 (0.37)	90.08
I trust the provider with my care.	4.80 (0.49)	84.30
I feel the front-office staff is courteous and respectful.	4.77 (0.48)	79.34
I would recommend this provider.	4.72 (0.69)	81.82
I feel the provider listens to me.	4.80 (0.51)	84.30
I feel I was seen by the provider in timely manner.	4.70 (0.74)	82.64
I appreciate the provider discussed illness prevention with me.	4.67 (0.68)	77.69
I feel I got enough information regarding treatments.	4.74 (0.60)	80.17
I feel my provider knows my medical history.	4.64 (0.71)	74.38
I feel my provider knows what to do if questions.	4.76 (0.56)	81.82

**Table 3:** Satisfaction by patient status Data are presented as N and mean (SD). A p-value < 0.05 is considered statistically significant. Abbreviations: N, number of patients; SD, standard deviation.

Score per question, lowest 1 to highest 5 (N = 121)	New (N = 46), mean (SD)	Returning (N = 53), mean (SD)	Post-op (N = 22), mean (SD)	P-value
I feel the provider shows respect to me.	4.87 (0.40)	4.87 (0.39)	4.95 (0.21)	0.6187
I trust the provider with my care.	4.83 (0.44)	4.74 (0.56)	4.91 (0.43)	0.3534
I feel the front-office staff is courteous and respectful.	4.74 (0.49)	4.79 (0.49)	4.77 (0.43)	0.8596
I would recommend this provider.	4.72 (0.62)	4.70 (0.67)	4.77 (0.87)	0.9133
I feel the provider listens to me.	4.78 (0.59)	4.77 (0.51)	4.91 (0.29)	0.5527
I feel I was seen by the provider in timely manner.	4.65 (0.77)	4.72 (0.66)	4.77 (0.87)	0.8075
I appreciate the provider discussed illness prevention with me.	4.59 (0.78)	4.64 (0.68)	4.91 (0.29)	0.1705
I feel I got enough information regarding treatments.	4.74 (0.61)	4.70 (0.64)	4.82 (0.50)	0.7365
I feel my provider knows my medical history.	4.54 (0.72)	4.64 (0.74)	4.82 (0.59)	0.3272
I feel my provider knows what to do if questions.	4.74 (0.53)	4.72 (0.63)	4.91 (0.43)	0.3871

**Table 4:** Satisfaction by pathology Data are presented as N and mean (SD). A p-value < 0.05 is considered statistically significant. Abbreviations: N, number of patients; SD, standard deviation.

Score per question, lowest 1 to highest 5 (N = 121)	Cranial (N = 55), mean (SD)	Spine (N = 66), mean (SD)	P-value
I feel the provider shows respect to me.	4.89 (0.31)	4.88 (0.41)	0.8583
I trust the provider with my care.	4.85 (0.40)	4.76 (0.56)	0.2838
I feel the front-office staff is courteous and respectful.	4.87 (0.39)	4.68 (0.53)	0.0284*
I would recommend this provider.	4.84 (0.60)	4.62 (0.74)	0.0858
I feel the provider listens to me.	4.82 (0.55)	4.79 (0.48)	0.7464
I feel I was seen by the provider in timely manner.	4.67 (0.88)	4.73 (0.60)	0.6872
I appreciate the provider discussed illness prevention with me.	4.80 (0.59)	4.56 (0.73)	0.0519
I feel I got enough information regarding treatments.	4.78 (0.57)	4.70 (0.63)	0.4427
I feel my provider knows my medical history.	4.75 (0.67)	4.55 (0.73)	0.1218
I feel my provider knows what to do if questions.	4.75 (0.62)	4.77 (0.52)	0.7920

**Table 5:** Satisfaction by number of providers Data are presented as N and mean (SD). A p-value < 0.05 is considered statistically significant. Abbreviations: N, number of patients; SD, standard deviation.

Score per question, lowest 1 to highest 5 (N = 121)	One provider (n = 107), mean (SD)	Two providers (n = 14), mean (SD)	P-value
I feel the provider shows respect to me.	4.92 (0.34)	4.64 (0.50)	0.0088*
I trust the provider with my care.	4.82 (0.49)	4.64 (0.50)	0.2018
I feel the front-office staff is courteous and respectful.	4.78 (0.48)	4.71 (0.47)	0.6537
I would recommend this provider.	4.73 (0.69)	4.64 (0.63)	0.6605
I feel the provider listens to me.	4.84 (0.44)	4.50 (0.85)	0.0180*
I feel I was seen by the provider in timely manner.	4.74 (0.70)	4.43 (0.94)	0.1402
I appreciate the provider discussed illness prevention with me.	4.72 (0.61)	4.29 (0.99)	0.0232*
I feel I got enough information regarding treatments.	4.76 (0.56)	4.57 (0.85)	0.2802
I feel my provider knows my medical history.	4.66 (0.67)	4.43 (0.94)	0.2439
I feel my provider knows what to do if questions.	4.79 (0.54)	4.50 (0.65)	0.0656

**Table 6:** Satisfaction by disposition Data are presented as N and mean (SD). A p-value < 0.05 is considered statistically significant. Abbreviations: N, number of patients; SD, standard deviation.

Score per question, lowest 1 to highest 5 (N = 121)	Follow up (N = 84), mean (SD)	Return as needed (N = 26), mean (SD)	Surgery scheduled (N = 11), mean (SD)	P-value
I feel the provider shows respect to me.	4.87 (0.40)	4.88 (0.33)	5.00 (0.00)	0.5467
I trust the provider with my care.	4.76 (0.53)	4.85 (0.46)	5.00 (0.00)	0.2842
I feel the front-office staff is courteous and respectful.	4.70 (0.53)	4.88 (0.33)	5.00 (0.00)	0.0566
I would recommend this provider.	4.65 (0.74)	4.81 (0.63)	5.00 (0.00)	0.2225
I feel the provider listens to me.	4.75 (0.58)	4.88 (0.33)	5.00 (0.00)	0.2019
I feel I was seen by the provider in timely manner.	4.67 (0.78)	4.69 (0.74)	5.00 (0.00)	0.3724
I appreciate the provider discussed illness prevention with me.	4.61 (0.73)	4.73 (0.60)	5.00 (0.00)	0.169
I feel I got enough information regarding treatments.	4.71 (0.61)	4.69 (0.68)	5.00 (0.00)	0.3099
I feel my provider knows my medical history.	4.57 (0.73)	4.69 (0.74)	5.00 (0.00)	0.1513
I feel my provider knows what to do if questions.	4.71 (0.59)	4.81 (0.57)	5.00 (0.00)	0.2562

**Table 7:** Disposition Data are presented as N and mean (SD). A p-value < 0.05 is considered statistically significant. Abbreviations: N, number of patients; SD, standard deviation.

Total number of patients (N = 121)		N (%)
Follow up		84 (69.4)
Follow up	Imaging needed	55 (65.5)
	Medical clearance needed	13 (15.5)
	Conservative therapies	12 (14.3)
	Records needed	4 (4.8)
Return as needed		26 (21.5)
Surgery scheduled		11 (9.1)

Next, our national commercial outpatient clinic patient experience survey responses from the neurosurgery clinic from 2022 to 2023 were obtained from the patient experience department at our hospital. Our hospital provided responses received from our neurosurgery clinic and also those from multiple neurosurgery clinics contracted with our national commercial outpatient clinic survey. The data reported over multiple years to our clinic from our national commercial outpatient survey combined all types of outpatient clinics. The survey responses were very low. Individual patient characteristics and outcomes were not available from the national commercial outpatient clinic patient experience survey to allow for direct comparison with our data.

## Discussion

At our institution, approximately 1,000 unique patients seen in the clinic can fill out the survey visits per year. Therefore, our national commercial outpatient clinic patient satisfaction surveys show responses from a very low percentage of the patient population. In contrast, real-time, in-person surveys had a higher response rate. In fact, our study shows a 100% response rate in all patients who were neurologically capable of filling out the surveys. The low response rate associated with our national commercial outpatient clinic patient satisfaction surveys has been previously reported and our data align with their finding [2,3]. One explanation may be multiple patients within the county system may not have adequate addresses and therefore may not receive the mail-in survey or be able to return it. Additionally, some of the patients who filled out the survey did not answer all the questions, hence differences in the number of responses per question.

The national commercial outpatient clinic patient satisfaction survey is a good tool and does provide a good estimate of the overall patient experience but fails to account for frequent changes address nuances and uniqueness of a clinic or answer specific questions. For example, it does not differentiate between types of patient status (new vs. returning vs. post-op), types of pathology (craniotomy vs. spine), types of providers (attending vs. resident vs. mid-level), specific providers at the time of surveys, and disposition (follow-up vs. scheduled for surgery vs. returned as needed). All of these are unique to a clinic and may not and should not be generalized locally and nationally; therefore, our national commercial outpatient clinic patient satisfaction survey does not include the information and only provides an estimate [5]. Our data also shows that our national commercial outpatient clinic patient satisfaction responses collected from different time frames differ from those from real-time surveys. Some responses are scored higher and others are lower; therefore, it is difficult to interpret the data and use it to provide feedback given a lack of specific days, teams, and patients on the national commercial outpatient clinic patient satisfaction survey. The lack of specifics is less helpful in training institutions where trainees need to learn how to modify behaviors in real time to improve patient interactions.

On the other hand, real-time, in-person, end-of-the-visit surveys bypass this generality. For example, our data show that there appears to be an improvement in the scores from new to returning to post-op status. This may be due to selection bias and/or familiarity with our providers. Patients seen by two different types of providers were less satisfied with “feeling respected by the providers,” “feeling listened to by the providers,” and “feeling appreciated that the providers discussed illness prevention.” Patients may prefer to be seen by the attending or a senior resident over a junior resident or a mid-level provider. They may not feel the ancillary provider is competent or have an inherent bias toward not being seen by a doctor. They may also fear that the information told to the ancillary provider is not being transmitted to the resident or attending properly. The extra time spent with two providers makes for a longer clinic visit and may have interfered with their schedules such as a return to work or same-day clinic visit in another specialty. The extra time spent with the ancillary provider may not be credited by the patient to that also spent with the attending; therefore, in the next study, an additional beneficial question maybe if the attending or another second provider spent sufficient time at the visit. Nevertheless, being mindful, respectful to patients, being active listeners, and being proactive in terms of discussing illness prevention are areas that can be improved.

The department reports are presented monthly, quarterly, semi-annually, or annually with corresponding benchmarks. Although the monthly reports provide attending names and their top answer scores and means, they do not account for a clinic that has rotating residents and mid-level providers who usually see the patients first. It therefore makes it difficult to assess which residents or mid-level providers interacted with the patients unless each attending only works with specific residents or mid-level providers at a given time frame. Moreover, at our institution, the publicly released national commercial outpatient clinic patient satisfaction responses are presented in a 6-month block. Each rotation is three months for residents, and it is variable for mid-level providers. This makes the responses obtained less helpful because it is unclear which behaviors need to be changed and which providers need to change them. Our national commercial outpatient clinic survey also does not account for behavioral changes that may be influenced by work-related stress or personal events.

Our national commercial outpatient clinic patient satisfaction survey provides useful information in clinics where personnel remain in constant attendance but does not provide individualized adequate real-time feedback. The surveys are mailed out days after the date of the clinic visit and the gap between clinic visit and survey completion may affect recalling their experience with the provider(s), especially if the patients have follow-ups with multiple specialties. They fail to provide real-time patient experience feedback. Real-time in-person surveys address this limitation and address the gaps not captured by national commercial outpatient surveys when the staff rotates. At the individual level, patient experience was immediately available for each provider and the feedback can modify behaviors in real-time. For example, if a patient disagrees that the provider discussed illness prevention during the clinic visit, this information was relayed to the provider and he or she became mindful to not forget to discuss illness prevention with other patients onward. At the collective level, overall feedback from all the patients seen on the same day is available for each rotating resident or mid-level provider. The responses were also evaluated on a weekly, monthly, or quarterly basis, just like our national commercial surveys but with specifics about the patients and providers. From specific provider-patient interactions to overall provider-patient interactions, the responses were invaluable for self-reflection, self-improvement, and behavior modification.

Overall, the low response rate, lack of specifics, and lack of real-time feedback associated with our national commercial outpatient clinic patient satisfaction survey made it difficult to interpret the findings provided by the patient experience department and to draw meaningful conclusions. On the other hand, we encourage the use of real-time, in-person, end-of-the-clinic-visit surveys to increase response rate and provide specifics to help improve patient experience and satisfaction level in real-time. Of note, the surveys were given at the end of the clinic visit and it was meant to not affect the encounter. Also, these surveys were filled out privately without the provider or another medical staff in the room to allow for genuine responses. This way the patients were not pressured to give better responses in fear of a compromise in their care.

There are some limitations of our study. This is a single-institution study. It is only conducted in the neurosurgery clinic setting and over a short period of time with a small sample size. Future studies should look at a larger sample size and a longer period of time. Our providers practice in multiple hospitals and it may be of interest to assess patient satisfaction in multiple neurosurgery clinics to evaluate for consistency. Another limitation is a lack of necessary information from our national commercial patient satisfaction surveys that prevented a direct comparison with the findings of our real-time in-person responses. It would be more insightful if this information were available. Modifications in how our national commercial surveys are delivered, collected, and analyzed may help provide information that is specific and useful and can be used to improve patient satisfaction.

## Conclusions

Patient satisfaction surveys not conducted in real-time and in-person run into the risk of low response rates, lack of specifics, and real-time feedback to help guide providers in quality improvement. Our data support the use of real-time, in-person patient satisfaction surveys to increase response rates and provide useful information to help improve patient experience. We therefore advocate for real-time, in-person surveys.
